# Real-world experience of Molecular Tumour Boards for clinical decision-making for cancer patients

**DOI:** 10.1038/s41698-025-00863-3

**Published:** 2025-03-25

**Authors:** Julio Herrero Colomina, Eleanor Johnston, Kate Duffus, Zoulikha M. Zaïr, Fiona Thistlethwaite, Matthew Krebs, Louise Carter, Donna Graham, Natalie Cook

**Affiliations:** 1 The Christie NHS Foundation Trust, Manchester, UK; 2 Division of Cancer Sciences, University of Manchester, Manchester, UK

**Keywords:** Genetic testing, Cancer genomics, Oncology

## Abstract

Molecular Tumour Boards (MTBs) play a crucial role in interpreting genomic results and providing treatment recommendations. We investigated the real-world impact of MTBs on clinical decision-making by surveying health care professionals (HCPs) across the UK; 44 participants from 11 MTBs took part in the study. 97.7% of respondents felt that MTBs increased awareness of available clinical trials matched to genomic alterations, 84% reported more confidence in interpreting genomic data, and 95.4% valued MTBs as educational. Hurdles to the discussion at MTBs included frequency and capacity of MTBs (ctDNA), sample collection and laboratory turnaround time (Tissue samples). One-third of respondents encountered challenges attending MTBs regularly due to workload. The survey highlighted areas for optimisation, such as meeting efficiency, rapid molecular analysis turnaround time, reliable trial matching tools, and ensuring MTBs are included in HCP's job plans.

## Introduction

Over the last decade, comprehensive genomic profiling has significantly advanced the field of oncology^1,2^. This progress has been driven by the discovery that certain genetic aberrations can predict how patients will respond to treatment. Consequently, multi-gene panels have been created to allow the testing of several aberrations at once, providing results in the most timely way and using the minimum amount of tissue. With the increasing availability and affordability of next-generation sequencing (NGS) the amount of data that Oncologists utilise to determine optimal treatment options for patients has increased considerably^1,3^. For this reason, a solid effort has been made to coordinate and standardise the reporting and interpretation of molecular alterations found in tissue and liquid biopsies^4^.

Precision oncology is the application of treatments tailored specifically for cancer patients, based on the findings from predictive biomarker analyses^5^. In recent years, the development of precision oncology and the complexity of genomic information has fostered the emergence of Molecular Tumour Boards (MTBs). These multidisciplinary teams aim to translate a vast amount of molecular data into clinically useful information^6–8^. Hamamoto et al.^9^ described the general workflow of Molecular Tumour Boards in six steps: assign biological significance to the genetic abnormality, interpret the genetic evidence for diagnosis and prognosis, attach specific candidate drugs and evidence corresponding to the genetic abnormality, discuss the significance of potential germline gene abnormalities, review the list of potential clinical trials according to the patient’s history and characteristics, and consider the patient’s condition for the selected candidate drugs.

Collaboration between clinicians and scientists is essential to fully evaluate the information available for each patient. The main aim of these multidisciplinary meetings is to provide a collaborative environment between physicians, geneticists, molecular biologists and bioinformaticians, amongst others, to interpret the molecular profile of patients together with the rest of their clinical information^2,10^. MTBs give specific recommendations to match patients with available treatments through the standard-of-care drugs, off-label therapies, or clinical trials^1,6,11^. An additional benefit of these meetings is that they represent a unique educational opportunity for all the involved professionals as well as medical trainees and students^11–13^. Since Kurzrock et al. first related their experience back in 2014^14^, MTBs have become a fundamental tool for modern oncology. These meetings are increasingly being used to support the complex clinical decision-making in precision medicine for patients diagnosed with cancer. MTBs are more than just platforms where experts uphold their specific domains of knowledge, they are ‘*genomic expertise in action*’^10^.

Since the establishment of the first Molecular Tumour Boards, our understanding of cancer biology has evolved at a rapid pace. Presently, we hold a vast amount of genomic data as well as clinical and pathological information which help us to better comprehend the unique situation of each cancer patient. Additionally, the therapeutic possibilities to link patients with targeted therapies have also increased significantly. As a result, several research centres worldwide have shared their experience regarding the feasibility and clinical benefit of these multidisciplinary meetings^1^. Globally, between 35.7% and 87% of patients referred to MTBs have actionable genetic alterations (mutations with therapeutic implications)^2,6,15–18^, and between 7% and 15% are enroled into matched targeted clinical trials^2,6,7,15,16^, although studies have shown that up to 41% of patients are also given genomics-based therapeutic advice^2,7,11^. These heterogeneous results reflect the variability in MTBs’ capacity to allocate patients to tailored treatments, the evolving knowledge regarding the cancer genomic landscape, and the different objectives of the published studies. However, the authors agree that MTBs constitute a critical tool for precision oncology. As an illustration, Miller et al. conducted a prospective phase II clinical trial in which authors demonstrated that MTB-directed therapy improved progression-free survival (PFS) over the immediate prior therapy in patients with advanced malignancies: they reported that the probability of PFS ratio (targeted therapy PFS/previous standard of care PFS) > 1.3 was 0.59 (95% CI 0.47–0.75)^18^.

Despite the abundance of data about Molecular Tumour Board outcomes, there is a paucity of information regarding the value of these multidisciplinary meetings perceived by the specialists involved in them. Bourret and Cambrosio underlined the expertise brought by the different professionals involved in MTBs^10^. VanderWalde et al. emphasised the importance of education to increase awareness of tailored therapies that match genomic alterations among oncologists^13^. Some studies have focused on the economic perspective: Luchini et al. stated that MTBs constitute 0.3% of patients’ total care costs^11^ whereas Walters et al. focused on cost savings where targeted therapies recommended by NGS vendors were not supported at MTBs. The predicted mean drug cost saved was $21,537 per month for each potential patient^8^. Moore et al. highlighted the utility of MTBs to determine if a referral to clinical genetics services is appropriate when an inherited genomic alteration is suspected^19^; in addition, they called attention to the lack of confidence among medical doctors regarding NGS interpretation. Authors have also raised concerns around other challenges, namely the delay brought by manual interpretation of NGS being a bottleneck for precision oncology^20^ or the insufficient experience of MTBs to draw conclusions regarding their clinical utility^6^.

**Fig. 1:**
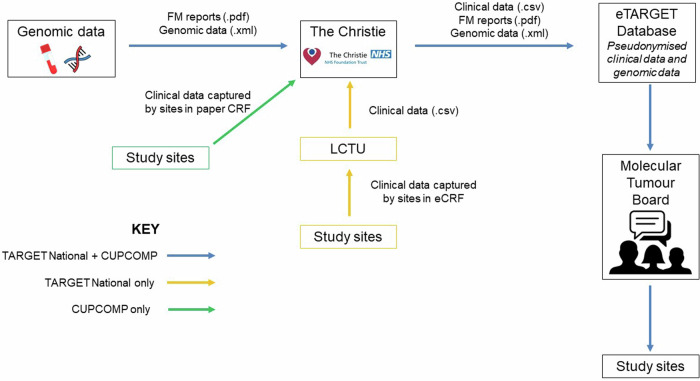
TARGET National and CUP-COMP dataflow diagram. CRF case report form, FM foundation medicine, LCTU Liverpool clinical trials unit.

**Fig. 2:**
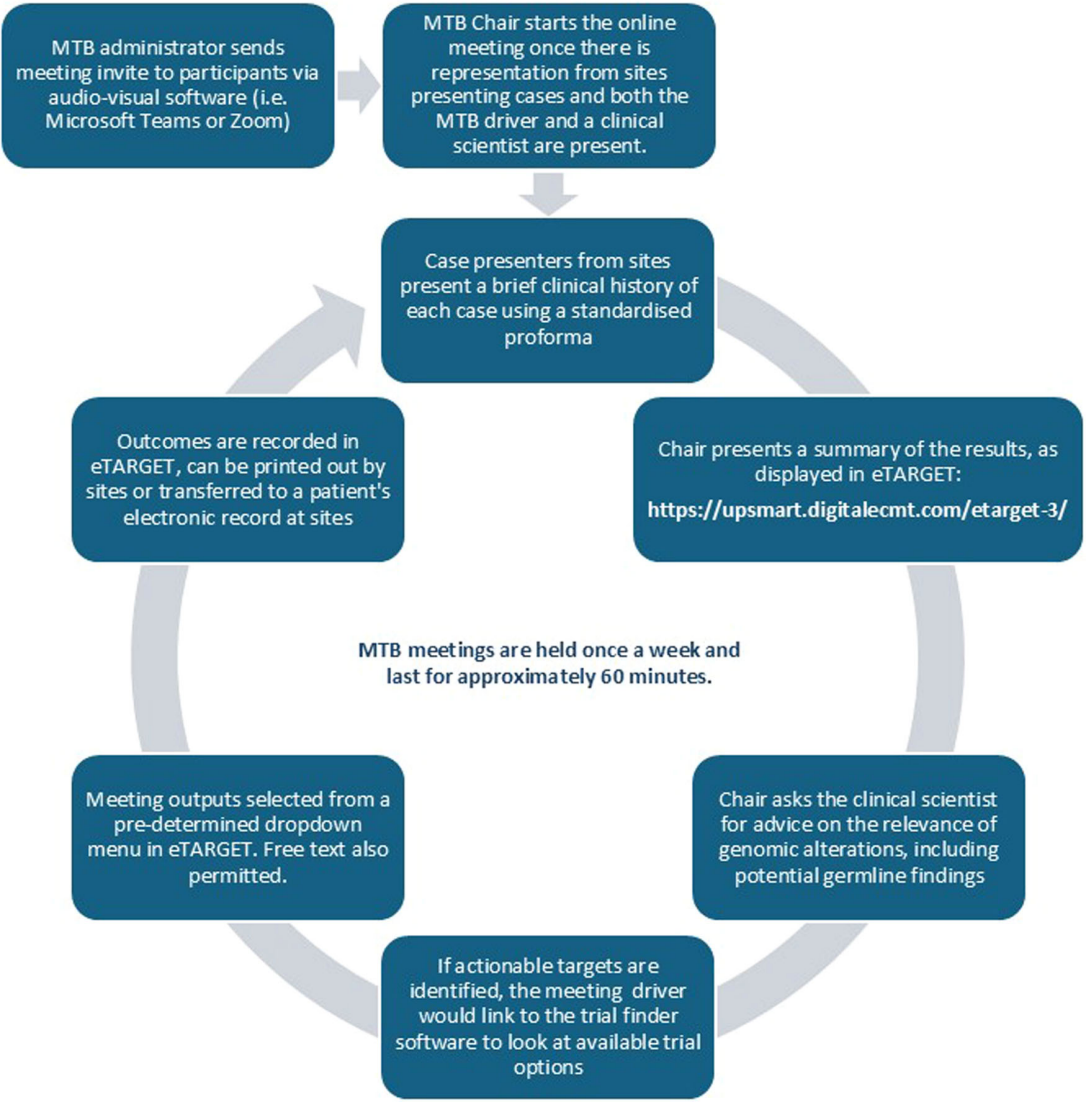
Flowchart of MTB meetings. MTB roles: Chair: A senior oncology professional with experience in molecular profiling, who moderates the meeting, ensuring that it runs effectively and coordinating the case discussion. Driver: A healthcare professional, generally a clinical fellow or a consultant, who navigates eTARGET during the meeting, looks for potential trials, and documents the MTB outcomes. Clinical Scientist: A healthcare professional in clinical genetics who reviews molecular profiling reports in advance of the meeting, determines the clinical significance of alterations and contributes to case discussions to identify actionable mutations. Case Presenter: A healthcare professional who prepares the clinical history of a patient and presents the case to the panel before the discussion. MTB Administrator: person in charge of setting the MTB agenda and sending meeting invites to participants. Also available if any technical issues.

This publication focuses on the implementation of MTBs for genomic interpretation within two investigator-led clinical trials in the UK, led by The Experimental Cancer Medicine Team (ECMT) at The Christie NHS Foundation Trust (Manchester, UK). TARGET National (NCT04723316) is a multicentre translational study that attempts to establish a national framework to offer molecular profiling to patients referred to Experimental Cancer Medicine Centres (ECMCs) in the UK, to assist in decision-making for allocation to molecularly targeted experimental cancer therapies^21^. Its two primary objectives are undertaking comprehensive genomic profiling on circulating tumour DNA (ctDNA) and/or tumour tissue (optional) in patients with solid tumours, and allocating patients within a National MTB to the most suitable matched experimental medicine therapies based on molecular and clinical characteristics^22,23^. Researchers across the ECMC network register patients to be discussed at the TARGET MTB after ctDNA or tissue genomic profiling has been analysed (Fig. 1). De-identified clinical data and genomic information is uploaded by researchers to eTARGET^24^, a specialised digital solution developed by the ECMT and the digital cancer research team in Manchester. This cloud-based software seamlessly integrates clinical and genomic sequencing data, facilitating virtual national discussions on MTBs. These expert meetings gather oncologists, health care professionals researchers and clinical scientists, who discuss patients’ results and provide feedback with regards to ‘actionable’ molecular alterations (See MTB workflow in Fig. 2). They use the online database ClinicalTrials.gov^25^ and two different digital trial matching tools, the ECMC experimental cancer (EC) trial finder^26^ and the Digital Cancer Research (DCR) trial finder^25,27^ to try to link patients with targeted studies. These are open-source digital tools that extract relevant study information from a corpus of clinical trials to support clinicians in matching their patients with precision medicine cancer clinical trials. Users can search using various criteria such as cancer type, molecular alteration, trial location or trial phase. Detailed information is provided regarding trial purpose, eligibility criteria and contact information for each study^25,27^.

The CUP-COMP trial (NCT04750109) is an exploratory, multicentre, clinical sample collection study that aims to make a comparison across tissue and liquid biomarkers in carcinomas of unknown primary (CUP)^28^. Its primary objectives are assessing genomic sequencing (both in tissue and blood) for the diagnosis and treatment stratification in patients with CUP, collecting evidence to further develop technology that predicts an individual’s response to a treatment and developing innovative systems of clinical data capture in patients with CUP^29^. It also utilises eTARGET to upload data and facilitate a national MTB for CUP (Fig. 1).

Assistance to interpret NGS and Whole Genome Sequencing (WGS) reports is also provided by NHS England Genomics Medicine Service and The Genomic Tumour Advisory Boards (GTABs). These are multi-disciplinary groups within the NHS that play a pivotal role in interpreting complex cancer genomic data emerging from the National Test Directory, and provide the link to local cancer multi-disciplinary teams which can guide patient treatments. Finally, there are numerous local MTBs that aim to assist the decision-making process in a regional setting.

For this study, we aimed to analyse the real-world experience of Molecular Tumour Boards in the clinical decision-making process for patients diagnosed with advanced cancer by evaluating our experience driving the TARGET National and CUP-COMP MTBs.

## Results

Forty-four participants (43.1% response rate) across the UK took part in the survey, out of which 42 (95.4%) were medical doctors (32 Medical Oncology Consultants and 10 Clinical Fellows), one (2.3%) nurse and one (2.3%) scientist. Table 1 outlines the MTBs attended by our respondents. Overall, 15 participants (34.9%) had been attending MTBs for <1 year, 19 (44.2%) for 1–3 years, 5 (11.6%) for 4–6 years, and 4 (9.3%) for more than 6 years.

**Table 1:** MTBs attended by survey participants

MTB name	Description of study/Background	Frequency of MTB	Genomics test used	References
TARGET National^a^ (NCT04723316)	Academic-led national study. Aims to match cancer patients with targeted early-phase clinical trials	Weekly MTBs held virtually	NGS of ctDNAon a blood sample and/or tumour biopsy(FoundationOne ® Liquid CDx and/or FoundationOne®CDX)	https://www.clinicaloncologyonline.net/article/S0936-6555(22)00489-7/fulltext
CUP-COMP^b^ (NCT04750109)	Academic-led national study. Compares the feasibility of molecular profiling from both tissue and blood in CUP patients	Monthly MTBs held virtually	NGS of ctDNA (FoundationOne® Liquid CDx) or tumour tissue (FoundationOne®CDX or WGS)	https://ascopubs.org/doi/10.1200/JCO.2024.42.16_suppl.3059
DETERMINE^c^ (NCT05722886)	Academic-led national study. Aims to test approved targeted therapies in unlicensed indications	Weekly MTBs held virtually	No genomic testing through this study – Patient’s samples undergo genomic testing at local Genomics Laboratory Hub (GLH) as per the National genomic test directory for cancer (https://www.england.nhs.uk/publication/national-genomic-test-directories/) or within other molecular screening programmes such as TARGET National	https://www.cancerresearchuk.org/about-cancer/find-a-clinical-trial/a-trial-of-targeted-treatment-for-rare-cancers-that-have-spread-determine
Genomic Tumour Advisory Boards (GTABs)	NHS-led standard of care advisory boards that discuss patients eligible for WGS	Regional virtual meetings	WGS as per NHS National genomic test directory	https://www.england.nhs.uk/genomics/
Precision-Panc^d^ (NCT04161417)	Master protocol. UK-wide platform for patients with known or suspected pancreatic cancer. Facilitates real-time profiling of pancreatic cancer patients for therapeutic and research purposes.	Virtual meetings held every 6 weeks	Illumina NGS technology. Targeted capture sequencing is carried out using the Glasgow Precision Oncology Laboratory (GPOL) Clinical Cancer Genome (CCG), a bespoke pancreatic cancer-specific multiplex assay accompanied by a purpose-built analysis pipeline (termed HOLMES), using publicly available and proprietary software developed in-house	https://www.clinicaloncologyonline.net/article/S0936-6555(19)30291-2/fulltext
Integrating Medically Actionable Genomics INto Early-phase trials (IMAGINE) (NCT42303887)	Glasgow ECMC-led project. Discussion of NGS reports from patients across Scotland to inform early-phase clinical trial selection.	Fortnightly MTBs held virtually	Illumina NGS analysis of tumours by in-house ‘Cancer-plus’ panel	https://www.hra.nhs.uk/planning-and-improving-research/application-summaries/research-summaries/imagine-2/ https://www.genomeweb.com/sequencing/university-glasgow-team-integrate-genomics-new-cancer-clinical-trials-network
Cancer Core Europe Basket of Baskets (NCT03767075)	Platform trial from Cancer Core Europe (CCE). Part 1 includes molecular profiling programme and an MTB. Part 2 includes a basket trial.	Weekly MTBs held virtually	CCE 350-gene NGS panel	https://basketofbaskets.eu/ https://ichgcp.net/clinical-trials-registry/NCT03767075 https://mtbp.org/

^a^Tumour Characterisation to Guide Experimental Targeted Therapy—National.

^b^Carcinoma of Unknown Primary: a comparison across tissue and liquid biomarkers.

^c^Determining Extended Therapeutic indications for Existing drugs in Rare Molecularly defined Indications using a National Evaluation platform trial.

^d^Precision-Panc Master Protocol: personalising treatment for pancreatic cancer.

### Value of the Molecular Tumour Boards

Nearly all the participants agreed that the implementation of MTBs had significantly increased their awareness of open trials to matched genomic alterations (97.7%), boosted confidence in interpreting genomic data and making precision medicine recommendations for patients (84%), and provided valuable educational opportunities for training future health professionals (95.4%). MTBs also encouraged collaborative opportunities between clinicians across the network (90.1%). Of note, 75% were not aware of evidence demonstrating the cost-effectiveness of MTBs.

### Patient selection for MTB discussion

Most respondents agreed that comprehensive molecular profiling should be offered to all patients being considered for clinical trial options (86.3%). The purpose of the MTBs was clear to 72.7% of respondents and 68.1% felt confident in deciding which patients should be discussed at the MTB. There was a difference in opinion when asked if all patients should be referred to the MTB after molecular profiling (38.6% agreed, 36.4% disagreed, 25% neither agreed or disagreed). Reasons for referring patients to MTBs included uncertainty about molecular interpretation (such as identifying the most relevant genomic alterations or clarifying complex results), seeking advice on trial options, interpreting rare molecular alterations for educational purposes, reviewing potential pathogenic germline variants, lack of treatment options, and needing details on available trials (i.e., cohorts, slots, inclusion/exclusion criteria).

### Delivery of MTBs

A significant proportion of professionals (63.6%) found it is easy to register patients for discussion at MTBs, 86.3% felt that the genomic information presented was in a clear format for decision making, 63.3% were satisfied with the frequency of the MTBs and the composition of the MTB in terms of expertise (79.5%). Noticeably, 34% of participants found it hard to attend the MTB on a regular basis.

**Fig. 3:**
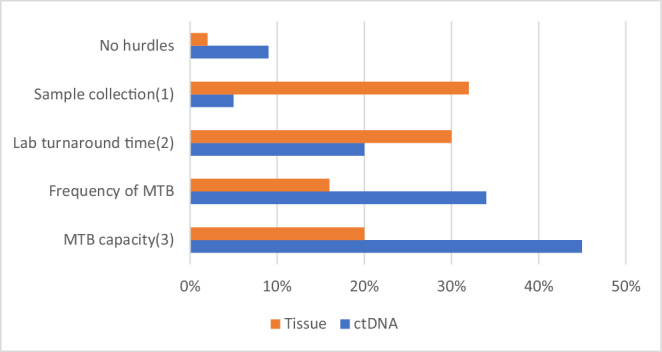
Participants’ perceived hurdles in obtaining prompt recommendations from MTBs. (1) Fresh tumour biopsy or archival tissue acquisition. (2) Genomics laboratory. (3) Extent to which the panel is able to review the cases, discuss them and provide recommendations.

The typical turnaround time from ctDNA acquisition to molecular analysis completion was under 3 weeks in 75% of cases, with time from blood sample collection to MTB discussion occurring within 2 months in all cases. While 34% found this period optimal for timely treatment decision, 25% disagreed. Figure 3 lists the main hurdles in obtaining prompt MTB recommendations, highlighting the differences between ctDNA and tissue samples.

For result interpretation, participants highlighted the value of fully informed presentation of patient cases for clinical decision-making. Notably, only 38.6% of respondents stated that there was always enough information to interpret the cases and make decisions in the MTB. When asked about which additional information alongside genomic data would enhance the MTB discussion, 47.7% mentioned pathology results, 40.9% wanted more detailed clinical data, 22.7% said radiology reports and 4.5% suggested complete family history. Overall, only 4 (9%) of participants stated there was not enough time to process the information in the MTB to make a recommendation to their patients.

### Trial matching through the MTB

The survey showed that 24 participants (54.5%) in this study had used the eTARGET platform for a Molecular Tumour Board. The benefits described by participants when using this system included ‘efficiency’ (in terms of having clinicopathological information and access to a trial finder tool in the same platform), ‘direct access to the trial finder’, ‘ease of recording patient information and accessing outcomes’, ‘virtual access’ and ‘immediate availability of the MTB results after the meetings’. However there were several limitations highlighted, including ‘lack of consideration of molecular pathways’, ‘inability to highlight Tumour Mutational Burden (TMB) or Microsatellite Instability (MSI) as clinically relevant’, ‘brief MTB outcomes’ and ‘delayed availability of NGS results’.

### MTB and trial matching software

Amongst our respondents, 24 participants (54.5%) used the EC trial finder, 16 (36.3%) used clinicaltrials.gov and 13 (29.5%) used the DCR trial finder. Respondents did mention the benefits of an integrated trial finder tool, discussing ‘the ease of access’, ‘its intuitive and time-saving design’, ‘the collaborative advantage of being integrated with the same platform as the MTB portal interface’, ‘the possibility of filtering the search results by different molecular alterations as well as trial location’, ‘the availability of the principal investigator’s contact details and the frequent updates’, among others. In contrast, participants were often unsure about the accuracy of some trial finders because they may find unsuitable trials as well as miss potentially relevant studies. Some participants claim that filtering for molecular pathways as well as specific mutations would help to increase the reliability of the trial finders. Additionally, respondents often required a trial finder tool that showed available slots for clinical trials in real time, which was not available for this study.

## Discussion

Molecular Tumour Boards represent an essential tool for modern oncology. The increasing amount of clinical, pathological and genomic data utilised in the management of patients makes these expert meetings a fundamental part of cancer diagnosis and treatment planning. MTBs represent a dynamic collaboration where collective expertise comes together to achieve meaningful outcomes for patient care. Our survey aimed to explore the real-world experience of MTBs as perceived by healthcare professionals based in the UK who participate in them. Most respondents believed that MTBs had deepened their awareness of pathogenic molecular alterations aligned to specific open clinical trials, a finding consistent with Kato et al.^30^. Following MTB participation, respondents also felt more confident when analysing genomic information and suggesting tailored treatment plans. These benefits from MTBs play a crucial role in shaping clinicians’ expertise and ensuring patients receive the best-personalised treatments.

Moreover, participants in our survey viewed MTBs as valuable educational platforms for future healthcare professionals and as catalysts for collaboration among clinicians nationwide and within networks. This value has been highlighted by several authors^12,13^ and represents a practical way of learning clinical genomics in action. Future efforts should focus on formalising MTB participation as part of medical education and continuous professional development.

A minority of respondents were aware of the cost-effectiveness of MTBs. This topic has been addressed in previous publications^8,11^, although remains a field that needs further exploration. A prospective study incorporating health economic evaluations, the economical implications of MTBs in healthcare, and their impact on patient’s outcomes, could facilitate the implementation of MTBs, alongside reviewing their cost effectiveness.

Most survey respondents advocated offering comprehensive molecular profiling with large cancer panels to all patients under consideration for clinical trials. However, there was disagreement regarding the referral of every patient to an MTB following molecular profiling. One of the main reasons why clinicians decide to refer patients to an MTB is the uncertainty in molecular interpretation^7,15,19^. It would be advisable to introduce tools such as the ESCAT framework (European Society for Medical Oncology Scale for Clinical Actionability of Molecular Targets)^31^ to categorise molecular targets in cancer based on the level of evidence supporting their clinical utility.

Accessible MTBs help oncologists better understand the complexity of the genomic alterations driving the patient’s cancer^6^. However, the work burden of MTBs is expected to increase in the coming years. As a result, there should be clear standardised criteria for guiding patient’s referrals to avoid exceeding capacity of these meetings.

Another reason for referring patients to MTBs was to review potential germline variants. This is supported by Moore et al.^19^, who stressed the usefulness of these multidisciplinary meetings in deciding whether to refer a patient to clinical genetics after identifying a potential inherited genomic alteration. The ESMO Precision Medicine Working Group published in 2019 their recommendations for tissue germline-focused analysis and genetics referral^32^. However, evidence regarding ctDNA germline analysis is scarce. MTBs open the door for referring possible cases of inherited cancer that may not have been previously recognised.

Most surveyed healthcare professionals found it easy to register patients for discussion in MTBs. They remarked that the genomic information presented during these meetings was in a clear format and facilitated decision-making. Additionally, respondents expressed satisfaction with the composition of the team in terms of expertise, similar to previous authors^10^. These results highlight the importance not only of the meeting itself but also of the digital tools that support it and the personnel that attend the meeting.

Notably, approximately one-third of participants encountered challenges attending MTBs regularly. This is understandable, given that most oncologists devote a significant amount of time to clinical duties and MTBs are not factored into most job plans. In-depth reviews of molecular data can be time-consuming and could limit the scalability of the MTB. Flexibility in scheduling MTBs, increasing their frequency, utilising support staff to prepare for MTBs, and extra funding to support the running of the MTBs would facilitate the running and participation in MTBs. There should also be recognition of MTBs in healthcare professionals’ job plans as part of their regular activities, in the same way MDTs are.

Participants reported a number of rate-limiting steps in obtaining prompt MTB recommendations. Sample collection and lab turnaround time stand out as the main challenges for patients who require tissue profiling. Other barriers included the frequency of meetings (and inability to attend) and MTB capacity. Some of these hurdles have been mentioned by researchers previously^20^ and future work should be done to lessen the impact of these barriers and minimise the time to obtain MTB recommendations. For instance, healthcare professionals could obtain extra administrative support for organising these meetings, and new digital tools could be incorporated to facilitate the MTB workflow. A summary of the MTB barriers and facilitators can be found in Supplementary Table 3.

Regarding result interpretation, some participants in the survey stated that they did not have enough data available to make a recommendation at the time of the MTB. Respondents felt that additional clinical data, pathology results, radiology reports, and further family history could enhance the discussion alongside genomic data. However, to do this would require extra administrative support, which is not always funded for MTBs. Therefore, MTB attendees must thoroughly prepare their clinical cases before the meetings to optimise the expertise brought by the multidisciplinary team. In addition, new advanced testing methods such as transcriptomics and proteomics are becoming more prevalent and hold significant potential for the selection of precision treatments for patients. Going forward, it will be important for MTBs to determine how best to incorporate emerging analyses such as transcriptomics and proteomics to streamline the conduct and preparation of meetings.

The survey results offer contemporary insights into the use of an electronic platform (eTARGET) integrated within a national MTB. The platform’s strengths, including its efficiency in integrating clinicopathological information with a trial finder tool, ease of patient information management, and immediate availability of MTB results, were highly commended. This tool is open sourced^24,33^. Other tools are available that incorporate these features and it is clear that integrating these tools into MTBs is useful to enhance workflow efficiency and facilitate timely, informed decision-making, which is crucial in the fast-paced environment of precision oncology. Participant suggestions, such as the addition of more detailed information on patients, and the development of a general framework to better understand MTB outcomes, indicate a need for enhanced data representation and clearer reporting to support more nuanced clinical decisions.

Finally, in terms of trial finder use, professionals appreciated the ease of access, user-friendly design, and integration of trial finders within the MTB platform, along with features like filtering by molecular alterations, trial locations, and access to principal investigator contact information. However, concerns were raised about the accuracy of trial finders, as some users encountered unsuitable or missed relevant trials. Some authors have emphasised the importance of establishing a link between MTBs and early phase trials teams to ensure the information on early phase trials and slots is available in real time^2^. This is especially challenging nowadays, taking into account the essence of dose escalation studies and the need for sponsor’s direct input on slots availability. Thus, the presence of a triallist in these meetings ensures an updated list of available slots for early-phase trials. Other possible facilitators include improving the trial finder’s filter to encompass not only mutations but also molecular pathways, designing country-specific trial finders and linking the MTB platform with different trial finder tools. Overall, while trial finders are valued, addressing these limitations could enhance their effectiveness in clinical practice^30^.

This study has limitations. Firstly, the survey response rate was <50% of healthcare professionals invited, but this is in keeping with response rates of other studies of this kind^34,35^. It was a study conducted across one health care system, and the respondents were already participating in MTBs as part of investigator-led trials, so this could skew the responses to the questionnaire, as many were already familiar with MTBs. The survey only included participants from secondary care hospitals, as this is where the vast majority of molecular profiling occurs in the UK. It would be very useful to understand global practice around MTBs, and also to survey a general oncology population, including clinicians not routinely involved in MTBs, to understand their perceptions and educational needs. Finally, our survey primarily utilised multiple choice and Likert scale questions to gather quantitative data, which, while providing a clear numerical representation of participant's perceptions and experiences, may not fully capture the depth and nuance of their insights.

Future complementary research incorporating more qualitative methods such as interviews or open-ended survey questions could help offer more detailed insights into the challenges, benefit and value of MTBs. This data could also help inform NHS policies in incorporation of genomics medicine as standard of care for patients.

In the era of precision medicine, Molecular Tumour Boards have emerged as a unique tool to assist clinical decision-making. Our study confirms the value of these multidisciplinary meetings perceived by healthcare professionals in matching patients diagnosed with cancer to targeted clinical trials, increasing clinician’s confidence in interpreting and actioning NGS results, creating a fruitful collaborative environment and serving as an incredibly useful educational opportunity for in-training doctors and healthcare professionals. This survey also shed light on the rate-limiting steps of MTBs and how to optimise the process in the future. More real-world studies are needed to compare the experience of MTB participants globally and improve their quality.

## Methods

### Study design and survey instrument

This study was approved by the Christie NHS Foundation Trust local Quality Improvement and Clinical Audit Committee on 6 April 2023 (reference 3574). A survey was distributed amongst the participants of the TARGET National and CUP-COMP MTBs held in the UK.

This survey aimed to explore the value of MTBs as perceived by healthcare professionals who participate in them (Supplementary Table 1). The objective was to better understand the impact of MTBs on medical decision-making, identify areas for improvement and develop best practices going forward.

Information gathered included participants’ perceptions of the MTBs clinicians attend, the average times between sample analysis and discussion at MTBs, and any rate-limiting steps in this process. We used a variety of question types to gather information, including free text responses, multiple choice questions, and presenting clinicians with statements related to aspects of the MTB and asking them to rate on a 5-point Likert scale. These were then grouped into themes across the MTB:

1. Value of the Molecular Tumour Boards
2. Patient Selection for MTB Discussion
3. Delivery of the MTB
4. Trial matching through the MTB
5. MTB and trial-finding software

The survey was sent electronically to 102 healthcare professionals involved in the TARGET National and CUP-COMP clinical trials and was open to responses from November 15 to December 15, 2023.

### Data analysis

A descriptive analysis was performed with the data gathered from the survey. Answers from free text questions were amalgamated and described if relevant.

## Supplementary information

Supplementary Information

## Data Availability

No datasets were generated or analysed during the current study.

## Code availability

No code was used in this study.
