# Supplementary material for: Real-world experience of Molecular Tumour Boards for clinical decision-making for cancer patients
Source: NPJ Precis Oncol. 2025 Mar 25;9:87. doi: 10.1038/s41698-025-00863-3 (PMC11937402; doi:10.1038/s41698-025-00863-3)
Supplement: Supplementary file 1 — Supplementary Information [file 41698_2025_863_MOESM1_ESM.pdf]

Supplementary data

Supplementary Table 1. Survey questions

Question no.	Question	Response type
1	What is your role?	Multiple choice
2	Which trust are you based in?	Multiple choice
3	I have attended MTBs for the following (please select all that apply)	Multiple choice
4	How many years of experience attending MTBs do you have?	Multiple choice
5 (Theme 1)	“The implementation of MTBs has increased my awareness of open trials to matched genomic alterations”	5 point likert scale
5 (Theme 1)	“MTBs have made me more confident in interpreting genomic data and generating precision medicine recommendations for patients.”	5 point likert scale
5 (Theme 1)	“I perceive MTBs to be an education opportunity for training health professionals for the future”	5 point likert scale
5 (Theme 1)	“MTBs have encouraged collaborative opportunities between clinicians nationally/ across the network”	5 point likert scale
5 (Theme 1)	“I am aware of evidence demonstrating the cost effectiveness of MTBs”	5 point likert scale
5 (Theme 1)	Any comments/suggestions?	Free text
6 (Theme 2)	“Comprehensive molecular profiling should be offered to all patients being considered for clinical trial options”	5 point likert scale
6 (Theme 2)	“All patients who have had molecular profiling should be referred to an MTB”	5 point likert scale
6 (Theme 2)	“The purpose of the MTB is clear”	5 point likert scale
6 (Theme 2)	“I am happy with deciding which patients should be discussed at the MTB”	5 point likert scale
6 (Theme 2)	Any comments/suggestions?	Free text
7	What factors do you consider when deciding whether to refer a patient?	Free text
8 (Theme 3)	For the following questions on delivery of the MTB, please answer with a specific MTB in mind (for example the MTB you are most familiar with). Please select the MTB of your choice below.	Multiple choice
9 (Theme 3)	“It is easy to register patients for discussion at MTBs”	5 point likert scale
9 (Theme 3)	“The genomic information presented is in a clear format for decision making in the MTB”	5 point likert scale
9 (Theme 3)	“I am happy with the frequency of the MTBs”	5 point likert scale
9 (Theme 3)	“I have time to attend the MTB on a regular basis”	5 point likert scale

9 (Theme 3)	"I am happy with the composition of the MTB in terms of expertise"	5 point likert scale
9 (Theme 3)	"The MTB is well resourced and funded to achieve its longer term aims/goals"	5 point likert scale
9 (Theme 3)	Comments:	Free text
Q10. What is the typical turnaround time for each of the listed activities in the MTB journey (from molecular testing to start of therapy)	Q10.1. Time to completion of molecular analysis – ctDNA	Multiple choice
	Q10.2. Time to MTB discussion – ctDNA	Multiple choice
	Q10.3. Time to completion of molecular analysis – TISSUE	Multiple choice
	Q10.4. Time to MTB discussion – TISSUE	Multiple choice
Q11. Turnaround times from molecular testing through to MTB recommendations	Q11.1. Turnaround times from ctDNA acquisition/molecular testing through to provision of MTB recommendations are currently optimum for timely treatment decisions	5 point likert scale
	Q11.2. Turnaround times from TISSUE acquisition/molecular testing through to provision of MTB recommendations are currently optimum for timely treatment decisions	5 point likert scale
	Q11.3. Optional comments?	Free text
Q12. If you think there are rate limiting steps in getting a timely MTB recommendation, what are they? (please select as many as apply)	ctDNA	Yes/No for a series of predefined options
Q12. If you think there are rate limiting steps in getting a timely MTB recommendation, what are they? (please select as many as apply)	Tissue	Yes/No for a series of predefined options
Q12	Do you have any additional comments?	Free text
13	Would additional pathology, radiology or clinical information alongside genomic data (WES, NGS, ctDNA) enhance the MTB discussion?	Multiple choice

14 (Theme 4)	"I always have enough information (clinical & genomics) to interpret the complex genomic and clinical information in the MTB to make decisions""	5 point likert scale plus comment box
15 (Theme 4)	"I always have enough time to process the information in the MTB to make a recommendation""	5 point likert scale plus comment box
16 (Theme 5)	Q16. Have you used the eTARGET tool for MTB?	Yes/No
17 (Theme 5)	Q17. What benefits does eTARGET bring to the TARGET National/CUPCOMP MTB? (please list up to 3)	Free text
18 (Theme 5)	Q18. Are there any limitations to eTARGET? (please list up to 3)	Free text
19 (Theme 5)	Q19. Would your site consider using eTARGET for other MTBs?	Yes/No plus comment box
20 (Theme 5)	Q20. What additional features /improvements could be added to eTARGET to optimise its function?	Free text
21	Do you use software/websites to look for eligible trials prior to the MTB?	Multiple choice
22	How frequently do you use these tools (% cases) - Integrated trial finder (within eTARGET)	Sliding scale 0-100
22	How frequently do you use these tools (% cases) - ECMC trial finder	Sliding scale 0-100
23	Please list up to 3 benefits of integrated trial finder (within eTARGET)	Free text
24	Please list up to 3 limitations of integrated trial finder (within eTARGET)	Free text
25	Please list up to 3 benefits of ECMC trial finder	Free text
26	Please list up to 3 limitations of ECMC trial finder	Free text
27	Would your site consider using the integrated trial finder (within eTARGET) for other MTBs?	Yes/No plus comment box
28	What additional features/ improvements could be added to the integrated trial finder to optimise its function?	Free text

Supplementary Table 2. Breakdown of participants

**Total number of participants**	44
**Participant's background**	
Medical Oncology consultant	32 (72.4%)
Clinical Fellow	10 (23%)
Nurse	1 (2.3%)
Scientist	1 (2.3%)
**Participant's experience on MTBs**	
Less than one year	15 (34.9%)
One to three years	19 (44.2%)
Four to six years	5 (11.6%)
More than six years	4 (9.3%)

Supplementary Table 3. Barriers and facilitators for the implementation of MTBs

	Barriers	Facilitators
Before the MTB	Delayed sample collection for DNA extractions (tissue and blood)	• Establish workflows for timely tumour/liquid biopsy acquisition and sample delivery to vendor<br>• Dedicated roles (translational research facilitators, pathology coordinators) to ensure timely sample collections
	Long turnaround time in laboratory to acquire the results and bioinformatic analysis	• Establish key performance indicators and target turnaround times<br>• Audit progress on a regular basis
	Increased workload due to MTB preparation	• Include administrative staff to help prepare meetings and ensure all reports are available before listing for MTB discussions<br>• Apply for extra funding/support from institutes or hospitals where meetings take place as core business
	Ability to interpret complex information from genomic reports	• Ensure clinical scientists have access to data at least 48 hours prior to meeting to prepare for the MTB
At the MTB	Difficulty attending the MTB on a regular basis	• Increase flexibility and rotate timings/days of meetings<br>• Recognise MTB in healthcare professionals' job plans<br>• Secure MTB accreditation as a recognized educational tool
	Insufficient MTB capacity	• Increase frequency of MTBs<br>• Streamline the meetings using available digital tools to efficiently<br>• Ensure all cases are prepared upfront and reviewed by the clinical scientists<br>• Chair to keep MTB discussions succinct and ensure meetings run efficiently
	Insufficient availability of comprehensive clinical and pathological information presented at the meeting	• Development of a proforma for case presentations at the MTB to include all relevant medical information<br>• Only present cases when there is a representative from the site the patient is registered to
	Inability to match a patient with a targeted clinical trial	• Establishing a link between MTBs and early phase trials' teams, with representative from these teams at the meetings<br>• Link MTBs with digital platforms and trial finder tools<br>• Improve digital tools such as trial finders to ensure they are country specific
After the MTB	Inaccurate record of MTB outcomes	• Develop a proforma to record the meeting outcomes in a systematic way<br>• Establish an MTB chair and a meeting driver that take minutes and register outcome<br>• Ensure outcomes reach the healthcare professionals presenting the case
	Lack of understanding of genomic reports	• Accreditation of MTBs as educational tools<br>• Use as educational opportunities for healthcare professionals' teaching
Others	Cost to healthcare of running the MTB	• Thorough health economic evaluation of cost effectiveness of MTB integrating into routine healthcare
